# Edge-Based Transfer Learning for Classroom Occupancy Detection in a Smart Campus Context

**DOI:** 10.3390/s22103692

**Published:** 2022-05-12

**Authors:** Lorenzo Monti, Rita Tse, Su-Kit Tang, Silvia Mirri, Giovanni Delnevo, Vittorio Maniezzo, Paola Salomoni

**Affiliations:** 1 INAF—Istituto di Radioastronomia, 40127 Bologna, Italy; lorenzo.monti@inaf.it; 2 Faculty of Applied Sciences, Macao Polytechnic University, Macao, China; ritatse@ipm.edu.mo (R.T.); sktang@ipm.edu.mo (S.-K.T.); 3 Department of Computer Science and Engineering, University of Bologna, 40126 Bologna, Italy; giovanni.delnevo2@unibo.it (G.D.); vittorio.maniezzo@unibo.it (V.M.); paola.salomoni@unibo.it (P.S.)

**Keywords:** Internet of Things, smart buildings, smart environments, deep learning, transfer learning, occupancy detection, smart sensing, ambient intelligence

## Abstract

Studies and systems that are aimed at the identification of the presence of people within an indoor environment and the monitoring of their activities and flows have been receiving more attention in recent years, specifically since the beginning of the COVID-19 pandemic. This paper proposes an approach for people counting that is based on the use of cameras and Raspberry Pi platforms, together with an edge-based transfer learning framework that is enriched with specific image processing strategies, with the aim of this approach being adopted in different indoor environments without the need for tailored training phases. The system was deployed on a university campus, which was chosen as the case study. The proposed system was able to work in classrooms with different characteristics. This paper reports a proposed architecture that could make the system scalable and privacy compliant and the evaluation tests that were conducted in different types of classrooms, which demonstrate the feasibility of this approach. Overall, the system was able to count the number of people in classrooms with a maximum mean absolute error of 1.23.

## 1. Introduction

The monitoring of the flow and presence of people, the counting of individuals in indoor environments (including means of transport) and the detection of whether people are present in a building or specific place have always been strategic goals within different contexts. In fact, such activities are able to provide information that can be useful and can be exploited for different purposes [1]. A few examples from the context of smart building management include the configuration settings of heat, ventilation and air conditioning (HVAC), alarms, lighting and building security systems [2].

After 2020, these monitoring actions have become vital. They represent a means to control and guarantee the realization of everyday activities in many contexts due to the need for social distancing in most public places, such as schools and universities, malls and stores, offices and workplaces, tourism and cultural entities and activities, etc., because of the ongoing COVID-19 pandemic [3]. In fact, social distancing has proven to be an effective measure for the reduction in contact between individuals and, consequently, the limitation of the spread of the virus [4].

In this context, the Internet of Things paradigm [5], together with the diffusion and availability of sensors and smart objects, can provide great support in the monitoring and detection of daily life activities within various situations. Examples include activitieshat are related to education and learning [6,7,8], those in the health and medical fields [9,10], those in the cultural heritage field [11,12] and other tourism activities [13], among many others [14]. Starting from the data that are collected and generated by this plethora of sensors, it is possible to train and then exploit intelligent algorithms to analyze the data, detect patterns or predict the occurrence of events.

In a previous work, a prototype for the detection of people in the indoor environments of a smart building was presented. That case study was particularly focused on classrooms on a university campus [15]. The use of camera-based approaches was investigated using an approach that was based on object-level analysis. Two different hardware solutions were compared: Microsoft Kinect and Intel RealSense cameras. The presented prototype was based on a client–server architecture and iteratively repeated the following operations: (i) the client, a Raspberry Pi 4 model B, took a picture of the people in a room using one of the two cameras that were evaluated; (ii) then, it sent the picture to a remote server that employed the HTTPS protocol; (iii) the server used the YOLOv3 model [16] to determine the number of people in the image; (iv) the result was stored on a MySQL database; and finally, (v) the image was deleted. The prototype was installed in a classroom on the campus that had a maximum capacity of 100 people. Two versions of the prototype were evaluated, both of which were pre-trained using ImageNet: the full version and a simplified version of the model. The experiments showed that the full version was able to achieve an accuracy that ranged from 85% to 92% while the simplified version did not work well, as demonstrated by accuracy values in the range of 20–40%. Both versions of the model worked better with images that were captured by the Intel RealSense camera. In [17], the architecture was refactored with the aim of improving the scalability and availability of the system. The possibility of moving the prediction layer from the server to the client was evaluated. This implied the use of the simplified version of the YOLOv3 model since the full version cannot be run on a Raspberry Pi 4 model B due to hardware constraints. Hence, the possibility of employing transfer learning was investigated by fine-tuning the weights of the model using a set of images that were taken within the classrooms on the campus. The system, which was only tested in a single classroom, proved to be effective for the detection of the occupancy of the classroom.

In this paper, we propose a further step to that research. The main contributions of this work consist of the proposed architecture and the transfer learning framework. They were especially designed for this case study, which had peculiar characteristics and requirements. First, the final architecture of our system is described, at the edge of which the classroom occupancy detection was performed, and the benefits of our architectural choices are detailed. Then, the approach for occupancy detection is described, which was based on transfer learning. Starting from the simplified version of the YOLOv3 model, which was pre-trained using the ImageNet dataset, the weights of the model were fine-tuned using images that were taken in two rooms of the university campus. Then, the results are presented, which were obtained by using the system in eight classrooms, each of which had different characteristics in terms of dimensions, capacity, orientation, position and lighting (e.g., window dimensions and position). Overall, the system was able to correctly identify the occupancy of the classrooms in this case study with an accuracy of over 90%.

The remainder of the paper is structured as follows. Section 2 briefly presents some of the main related work and compares the approaches that can be adopted for similar purposes. Section 3 describes the proposed architecture of our approach and Section 4 presents the employed methodology, with details of the dataset and the training process that were adopted. Section 5 illustrates the results that were obtained during the testing phase, with a focus on the system performance in terms of accuracy in detecting the presence of people and counting the number of people in the indoor environments. Finally, Section 6 concludes the paper with some final remarks and discloses the main areas for future works.

## 2. Background and Related Work

This section briefly presents some of the main background and related works, with a focus on the most interesting approaches that have been used for occupancy detection systems in indoor environments. Over the years, this particular field of research has been extensively investigated, as demonstrated by several surveys [18,19,20,21]. Such systems have vast applications, the most common of which consist of resource optimization, such as the optimization of energy consumption, the control of HVAC equipment and the management of lighting systems, and user comfort, which includes the quality of indoor environments and personal thermal comfort systems. Other possible applications include intelligent transportation systems, surveillance systems and healthcare and health monitoring.

Occupancy detection approaches can be divided into two main categories: image-based vs. non-image-based. Non-image-based techniques are approaches that do not leverage image or video streams and instead take advantage of different technologies and architectural solutions. The first family of approaches relies on passive infrared (PIR) sensors [22,23]. Human bodies constantly emit heat in the form of radiation, which occurs at the infrared wavelength. Even when outside of the visible range, PIR sensors can detect the radiation that is emitted by people who are within the field of view of the sensors. The main advantage of PIR sensors is their low cost; however, they present many limitations, which include low reliability since they require a clear line of sight and, more importantly, the people to be in continuous motion. Finally, they can indicate the presence (or absence) of people but not the number of people. These limitations can be partially mitigated by using other sensors or multiple PIR sensors that are placed on the top of doorways, for example [24]. A second family of approaches exploits ultrasonic sensors. These devices transmit wideband ultrasonic signals and process the superposition of reflected received signals [25]. Contrary to the PIR sensors, these require neither a clear line of sight nor the continuous motion of the people. However, as for PIR sensors, they can only determine the presence (or absence) of people [26]. A further family of approaches employs tag-based sensors, such as radio frequency identification (RFID) technology [27]. The main components of these systems are tags, tag readers and middleware. These approaches can provide information that is related not only to the presence of people but also to the location and number of people. The number of people is often computed using some form of probabilistic estimation [28]. The main limitations of these approaches concern the sensitivity of the system, which is affected by certain environmental conditions, such as humidity [29], and the requirement for additional hardware. The next family of approaches is based on Wi-Fi access points. Since devices such as smart phones and laptops use probe request frames to scan areas in order to identify WLAN networks, they are able to estimate the number of people within an area who are covered by the same access point [30]. The main advantages of this type of approach are the use of infrastructures that already exist and the preservation of the privacy of users. The main drawback is that people with multiple devices may be counted more than once while people who are in the area but are not connected to any network activity cannot be counted [31]. Another family of approaches is based on carbon dioxide (CO_2_) sensors [32]. CO_2_ is produced by the human respiratory system; hence, the measure of CO_2_ in indoor environments can be an indicator of occupancy [33]. The major limitation derives from the fact that, since the dispersion of gas is a slow process, the sensors have slow response times [34]. Furthermore, the concentration of CO_2_ is significantly affected by the outdoor environment. To mitigate these issues, these sensors are often coupled with other environmental sensors [35,36].

In general, non-image-based techniques have several advantages, such as low costs, the fact that they are privacy compliant and they often require a minimal infrastructure in order to work. However, most of these techniques are only able to detect the presence (or absence) of people in an indoor environment while only a few of them are capable of counting people and even they have low rates of accuracy. For this reason, we focused on image-based approaches that were based on images or video segments, which were captured by a digital camera. Camera-based approaches are able to achieve higher levels of accuracy in the detection of the occupancy of indoor environments than non-image-based techniques. The main drawbacks are their costs and privacy concerns, i.e., cameras can be expensive compared to the other types of sensors and adequate data pipelines are necessary in order to respect the privacy regulations of different countries. There are two main methods for the automatic counting of people in images. One method is called line of interest (LOI) and works on a temporal slice of a video [37] to count the number of people who cross a virtual line of interest within the monitored scene [38,39,40]. This method can estimate both the cumulative count (i.e., the total count of people since the beginning of the video) and the instantaneous count (i.e., the count at any given time). The second method is called region of interest (ROI), which can estimate crowd density by evaluating the number of people who are present within a specific region of interest in the monitored scene [41,42,43].

Since we were interested in counting the number of people in the different classrooms and laboratories on a university campus, we focused on ROI methods. Depending on the main strategy of analysis that is employed, ROI methods can be grouped into three main categories:

1. Pixel-based analysis: the methods that are based on this analysis are more focused on the estimation of density rather than whole counts. They extensively use local features, such as edge information or individual pixel analysis, to count [44,45,46];
2. Texture-based analysis: this analysis represents an active and interesting topic within image processing and plays a significant role in many applications, such as image retrieval and face recognition. It relies on texture modeling through the analysis of image patches [46,47]. Among the texture-based analysis methods, there are gray-level co-occurrence matrices, Fourier analyses and fractal dimensions [48];
3. Object-level analysis: the methods that are based on this analysis try to locate different types of objects within a scene by first determining whether objects that correspond to the correct classes are present in the scene and then finding where they are placed in the scene [44,49,50].

In this work, a ROI method that was based on object-level analysis was adopted. In a previous work [15], we presented a prototype and compared the system using two different cameras in a single classroom: a Microsoft Kinect and an Intel RealSense. Then, in [17], we improved the system architecture and analyzed its performance in two classrooms. In this work, we present the results that we obtained by employing the YOLOv3 model. In particular, we used a YOLOv3 model that was pre-trained using the ImageNet dataset and then, we applied transfer learning in order to fine-tune the model for counting people in a classroom. We then tested the system in eight classrooms on a university campus.

## 3. Our Proposed Architecture

In this section, the system design of our platform is presented by detailing the entire architecture, which was composed of a client side and a server side, each of which contained different layers. In our first experiments [15], a common client–server architecture was used in which the computation was performed by the server side. This solution only worked in scenarios with few devices and it could not be scalable in any way. In this sense, we aimed to change the weights of our architecture (*fat client–thin server*) by shifting the computation to a client-embedded device: a design choice that was well supported by current architectures, even for computationally demanding tasks [51,52], while maintaining a good level of accuracy for the prediction of the number of people in a classroom [17]. In this way, this method is expected to produce many benefits:

- Higher scalability: fat clients can complete jobs independently from other clients and then send their results to the server;
- Working semi-offline: in this way, it is possible to predict the number of people in a scene and store that result directly on a single-board computer without the need to send the data immediately;
- Higher availability: instead of having a single point of failure, there are different clients that work independently. This allows the system to be more robust;
- Privacy compliant: the number of people and the time at which the frame was analyzed are the only data that are stored in the client node and sent to the server side.

**Figure 1:**
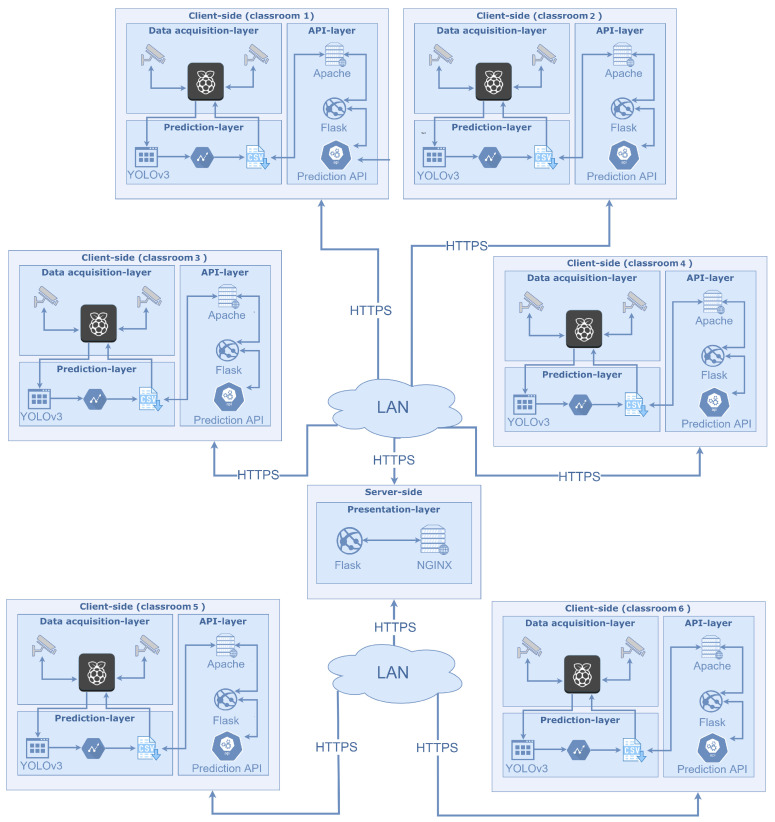
The fat client–thin server architecture that is being proposed. The server side is located at the center of the image, while the client nodes are located around the edges.

The proposed architecture is presented in Figure 1. It is important to highlight that almost all of the computation occurs on the client side. Each layer of the architecture is discussed in isolation in the following subsections.

### 3.1. Data Acquisition Layer

This layer was devoted to data acquisition, with a focus on the monitoring of the occupation of classrooms and laboratories and the comparison of their capacities. In order to count the number of people in an indoor area, two different kinds of low-budget cameras were compared:

- *The Intel RealSense D415 Depth camera:* RealSense technologies provide a suite of depth and tracking technologies, which make it possible to count the number of people within a given area. This camera is USB-powered and consists of an infrared projector, a pair of depth sensors and an RGB sensor. The depth output resolution can be set at up to 1280 × 720 pixels and the depth frame rate can be set at up to 90 fps. The RGB frame resolution is 1920 × 1080 pixels and the maximum frame rate is 30 fps. For this case study, the camera was plugged (via USB) into a Raspberry Pi 4 model B and 1280 × 720 pixel frame images were acquired every five minutes (we set this time interval in order to better support the storing operations);
- *The Microsoft Kinect camera:* initially, the Kinect was developed as a gaming tool for Xbox 360. It contains three main components that work together to detect the motions of the user and create their physical image on the screen: an RGB color VGA video camera, a depth sensor and a multi-array microphone. As for the camera, both the video and depth sensor have a 640 × 480 pixel resolution and run at 30 fps. This camera was also plugged (via USB) into the Raspberry Pi 4 model B and 640 × 480 pixel frame images were acquired every five minutes.

After performing the accuracy tests for each low-budget camera, as explained in our previous work [15], the Intel RealSense D415 Depth camera was chosen. The selected camera, which was devoted to the prediction phase, acquired RGB images before applying our custom deep learning model. Depending on the classroom size, either one camera (for *small* classrooms) or two cameras (for *large* classrooms) were installed.

### 3.2. Prediction Layer

The prediction layer retrieved data from the cameras on the client side and exploited a custom model that was based on YOLOv3 [16], with the aim of detecting the number of people within the images. This tool applied a single neural network to the full image. Specifically, this network divided the image into regions and predicted the bounding boxes and probabilities for each region. The bounding boxes were weighted by the predicted probabilities. The model reported certain advantages over classifier-based systems. It considered the whole image at the given test time, with the aim of allowing the predictions to be informed by the whole context, as depicted in the picture. This library could perform predictions using a single network evaluation, unlike systems such as R-CNN, which require thousands of evaluations for a single image. This makes our method extremely fast: approximately more than 1000× faster than R-CNN and 100× faster than Fast R-CNN [16]. Once the prediction was complete, the number of people that were detected and the timestamp at which the input image was taken were saved in a CSV file.

### 3.3. API Layer

Each client was exposed to the same set of APIs, which could be queried by the server in order to retrieve the number of people that were detected by each camera within a specific period of time. The communication between the clients and the server took place over HTTPs. As already anticipated, these architectural choices guaranteed the scalability of the overall system and also allowed the clients to work offline.

### 3.4. Presentation Layer

The presentation layer was the only layer that was present on the server side. It interacted with the API layer to retrieve data concerning the classroom occupancy and visualize those data. It was implemented as a web application using standard web technologies, such as HTML5, CSS3, Javascript, etc. The back-end system was developed using *Flask*, which is a Python micro-framework. Lastly, *NGINX* was employed as a web server and reverse proxy in order to make the pages available on port 80 and also serve static files.

Due to this layer, administration staff could have an overall view of the occupancy of all classrooms across the campus. Even though this system currently only provides some basic visualizations, it could also be enriched with advanced analytics.

## 4. Methodology

**Figure 2:**
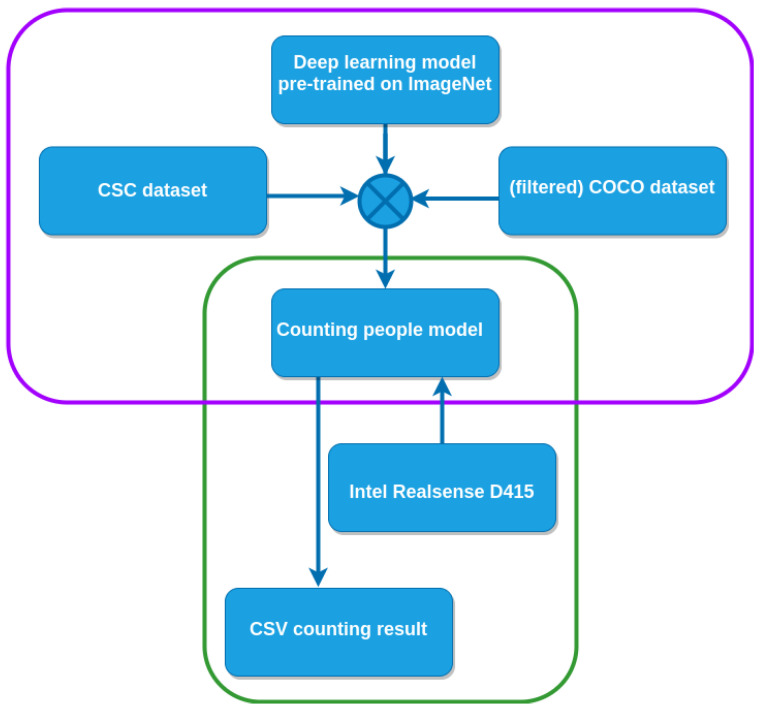
The proposed transfer learning framework. The model, which was pre-trained using the ImageNet dataset, was fine-tuned using the CSC and filtered COCO datasets. Once deployed, the model counted the number of people who were present in the images that were captured by the Intel RealSense D415 cameras.

In this section, we present our proposed methodology for counting the number of people within the context of a smart campus, with our previous studies being used as a starting point for this work [15,17]. The experimental setup consisted of eight classrooms on the Cesena campus of the University of Bologna. The classrooms were different in size, layout, number of seats and orientation. For each classroom, a client node was set up and, depending on the classroom size, it was equipped with either one or two cameras. The main steps of the proposed methodology, which was based on transfer learning, are depicted in Figure 2. As shown, the proposed approach was composed of two major steps. In the first step, a deep learning algorithm that was pre-trained using the ImageNet dataset was then trained using two specific context datasets for the classroom occupancy detection task. One of these two datasets, the *Classroom Student Counting (CSC)* dataset, was collected using two of the eight cameras that were installed on the campus. The second was a portion of the COCO dataset [53]. Upon the completion of the training process, the system was ready to count people in the images that were captured from the Intel RealSense D415 cameras, which comprised the second step of proposed methodology, as depicted by the green rectangle in Figure 2. The number of people that were detected in the room was stored in the client node and could be retrieved by the server using the API that was exposed by the client, as described in the previous section.

### 4.1. Dataset Description

As previously mentioned, we took advantage of two different datasets. The CSC dataset was the result of the data collection process that employed the experimental setup that was installed on the Cesena campus. The data were collected during different sessions and involved volunteer students. In all sessions, the volunteers signed a release form that was privacy compliant and then occupied the classroom as though they were attending a lecture. The students sat in different positions around the room with the aim of collecting different configurations of classroom occupation. Only two of the eight classrooms were used to collect the training dataset. In this way, during the testing phase, the model could be evaluated using data from the classrooms that were not used during training. Specifically, one classroom that was equipped with only one camera (a *small* classroom) and one that was equipped with two cameras (a *large* classroom) were selected for the training dataset, which contained 1196 and 808 images from the small and large classrooms, respectively.

Even though the manual annotation of a huge amount of data requires a lot of work, it is essential for supervised learning methods. The labeling process was as follows. First, we took advantage of a pre-trained YOLOv3 model. In this way, it was possible to roughly retrieve most of the bounding boxes for the *Person*, *Chair* and *Backpack* classes. Then, possible errors that were made by the model when recognizing the three classes were manually corrected. Finally, the *Jacket* label was added to all images. We decided to also identify backpacks and jackets with the aim of minimizing the number of potential false positives (i.e., backpacks or jackets that were misidentified as people).

**Figure 3:**
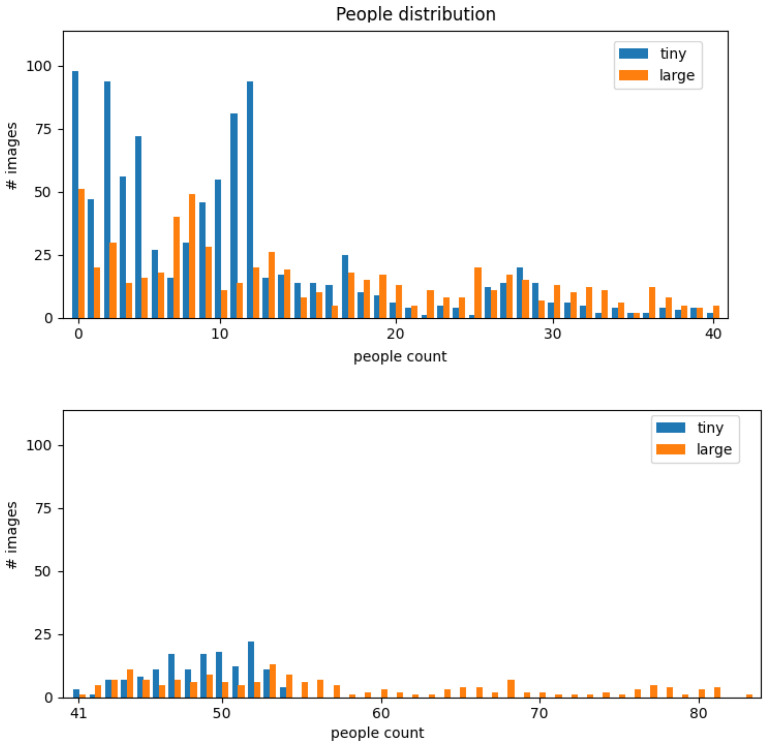
Distribution of the number of people that were counted.

Hence, the Classroom Student Counting (CSC) dataset consisted of four classes: *Person*, *Jacket*, *Chair* and *Backpack*. In Figure 3, the distributions of people in the images for both the small and large classrooms are plotted. The upper chart depicts the number of images that contained 0–40 people, while the lower chart details the number of images that contained 41–83 people. Specifically, for the small classroom, the number of people ranged from 0 to 54, with a mean of 13.6 and a standard deviation of 15.7, while for the large classroom, the number of people ranged from 0 to 93, with a mean of 23.7 and a standard deviation of 20.8.

With regard to the distribution of the Jackets class, the number of jackets ranged from 0 to 14 in the small classroom, with a mean of 1.7 and a standard deviation of 1.9, while the number of jackets reached 83 in the large classroom, with a mean of 23.7 and a standard deviation of 20.8. For the distribution of the Backpacks class, the number varied from 0 to 11 for both classrooms, with a mean of 2.6 and a standard deviation of 2.4 for the small classroom and a mean of 4.1 and a standard deviation of 2.5 for the large classroom.

Finally, the COCO dataset [53] was also used. This dataset was initially released by Microsoft for the detection and segmentation of objects that are found in natural environments in everyday life. The cumulative release in 2015 contained 165,482 training images, 81,208 images for validation and 81,434 testing images. The images were relative to 91 object types, including our four objects of interest. Since the dataset was already labeled, only the images that contained one of the four classes of interest were selected, with a total of 67,316 images.

### 4.2. Training Process

Once the dataset was ready, the training process began. First, the dataset was divided into training and validation sets, which used 70% and 30% of the data, respectively. A portion of the data was not kept aside for the testing phase since the model was directly evaluated online. The data were divided based on the number of people that were present in the images, so as to maintain the same proportion in both sets. In terms of the model, YOLOv3 [16] was employed, particularly its implementation of darknet [54]. As previously stated, we started with a pre-trained version, *darknet53.conv.74* (https://pjreddie.com/darknet/yolo/, accessed on 15 December 2021),

Which contained the weights of the darknet network that were originally used to train the classification using the ImageNet dataset, which was in turn used as the pre-trained feature extractor (backbone). In the last layer of the network, which was fully connected and output the class probabilities of classification, the weights were randomly initialized prior to training. The size of the input images was set to 608 × 608 pixels and the learning rate was equal to 0.001.

Then, the training process was launched, using both the filtered COCO dataset and the CSC dataset. The number of epochs was a crucial hyper-parameter to set: it had to be high enough to ensure that the model was able to correctly detect people (avoiding the problem of underfitting), but a number that was too large could cause the opposite phenomenon of overfitting (in which the model adjusted to some random relation that only existed in the dataset and not in the real world examples) and hence, lose the ability to generalize and operate adequately with new images. The number of training epochs was set to 105,000, with a checkpoint at every 1000 epochs to restore the weights of the given epochs.

**Figure 4:**
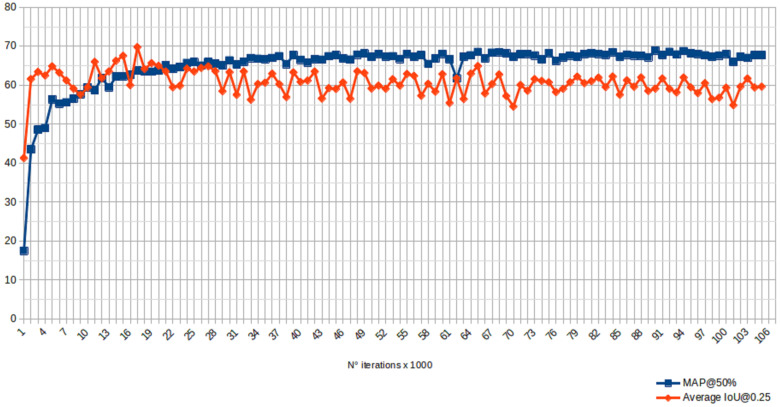
Average IoU and mean AP, which were computed using the validation set during the training process.

Two metrics were evaluated every 1000 iterations during the training process. Object detection systems make predictions that are based on bounding boxes and class labels. For each bounding box, the system measured the overlap between the area of the predicted bounding box and the area of the ground truth bounding box. So, the first metric was measured using the intersection over union (*IoU*). The precision and recall are also often calculated using the IoU value for a given *IoU* threshold, which was the average *IoU@0.25* in our case. The second metric was the mean average precision *mAP*. This metric is based on the average precision that is related to the area under the precision–recall curve of a given class and is calculated as:

$$AP=\int_{1}^{0}p\left(r\right)dr$$

where *p* and *r* are the precision and recall coefficients, respectively. The process was iterated over each class and then averaged. A confidence threshold parameter of 50% was chosen. Figure 4 depicts the curves of the two metrics (MAP@50% and the average IOU@0.25), along with the different iterations of the training process.

The values of the two metrics are also presented in Table 1. In addition to mAP and IoU, the relative precision, recall and F1 score values are also reported. For the sake of conciseness, only the values of the metrics at every 5000 iterations are reported in the table. Based on the selected evaluation metrics, the best result was obtained at iteration 65,000.

## 5. Results and Discussion

**Figure 5:**
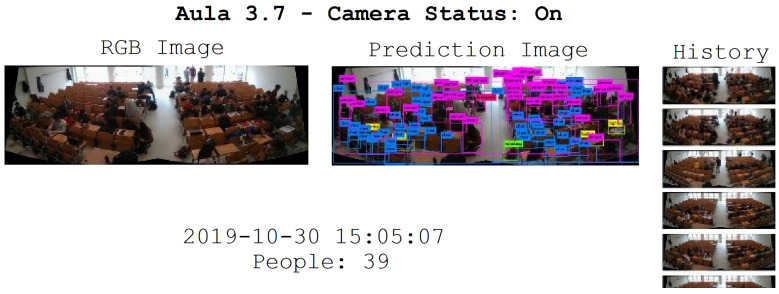
An example of an acquired image, with the prediction and labeling of people, jackets, backpacks and chairs (two cameras; large classroom).

In this section, the results that were obtained during the testing phase are explained. As already mentioned, the accuracy of the model was evaluated using a set of heterogeneous images, which was not used during the training (or validation) phase. As described in Section 3, two different types of classroom were used in this experiment (small or large), with a total of eight classrooms (five small and three large). The images in the CSC dataset were collected using small classroom #1 and large classroom #1. To test the accuracy and overall correctness of our system, one hundred images were acquired from each camera in all of the classrooms (100 frames from small classrooms and 200 frames from large classrooms), with a total of 1100 images. The images were acquired with several volunteer students varying their positions within the classrooms (an example of an acquired image and the related labeling is shown in Figure 5). The data collection process for each classroom was carried out at different times, in different seasons and under different weather, temperature and lighting conditions.

Different evaluation metrics were used to evaluate the test dataset. First, the accuracy of the system in the detection of individuals was evaluated. Three metrics were considered:

- Real number (RN): the exact number of people who were present at the time of the image, as counted by a human operator;
- False counting number (FCN): the errors that were made by the system, such as situations when a person was counted twice as a result of the person’s movement or when the print on a t-shirt was counted as a face;
- Predicted number (PN): the number of people that was predicted by our customized YOLOv3 model.

To assess the accuracy of the proposed people counting system, the following formula was also used:

(1) $$\mathrm{Accuracy}\left(\%\right)=\left\{\begin{array}{cc} \frac{\mathrm{PN}-\mathrm{FCN}}{\mathrm{RN}}*100,\; & \forall \;\mathrm{PN}\leq \mathrm{RN}\\ \frac{\mathrm{RN}-\mathrm{FCN}}{\mathrm{RN}}*100,\; & \forall \;\mathrm{PN}>\mathrm{RN} \end{array}\right.$$

Then, the root mean square error (RMSE) and the mean absolute rrror (MAE) were also computed by contrasting the RN and PN.

All of these metrics are reported in Table 2. Since the accuracy was calculated as the average of the accuracy across all images, the standard deviation is also reported. As shown, the system was able to detect classroom occupancy with a high level of accuracy overall, as demonstrated by the high values of accuracy and the low values of RMSE and MAE. It is interesting to highlight that the system performed better in small classroom #1 and large classroom #1, which were the classrooms that were used to collect the CSC dataset and were then also used during the training phase.

Moreover, it can be seen that the average accuracy values were lower in the large classrooms than the small classrooms, while the standard deviation values were lower in the small classrooms than the large classrooms. The reason for this seemed to be the higher complexity of the task (class occupancy detection) in the large classes, which are used for university courses that have lots of students because they have more seats. Another factor that could have influenced the lower performance of the system in large classrooms was the position of the two cameras. The average accuracy and the related standard deviation were calculated by splitting the frames per camera inside each large classroom. The results are presented in Table 3. As shown, in general, there were no significant differences in performance between the two cameras.

**Figure 6:**
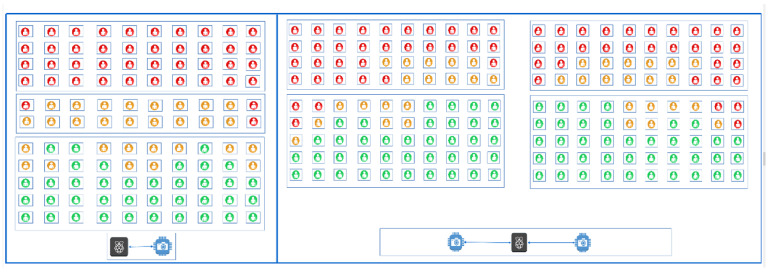
Critical areas for the occupancy detection system in both small and large classrooms.

Focusing intently on both small and large classrooms, we analyzed the errors that were made by the system and paid particular attention to the false positive results. Most of the errors were produced by over-counting when more than 30 students were present in a classroom. By looking at the frames that were used for our tests, it was possible to notice the critical area and that it corresponded to the back rows of the classroom, as shown in Figure 6. In that area, which ranged approximately between eight and twelve meters away from the camera, the bounding boxes were very small and the people also appeared closer together. To solve this issue, the dataset could be expanded with ad hoc examples or different strategies could be adopted to count the people in the back rows of classrooms.

Overall, the system proved to be effective for the task of classroom occupancy detection within a smart campus context. The proposed architecture provided several benefits, such as scalability and availability, the possibility of working in a semi-offline fashion and low-cost equipment; moreover, this proposed approach was privacy compliant, according to the current regulations. It is worth noting that an approach such as this could be useful in many different contexts, ranging from the more effective planning of classroom and lab management to the monitoring of people density so as to avoid possible overcrowding situations, which support the actions that are devoted to limiting the COVID-19 pandemic. Other contexts in which this approach could be useful include the strategic counting of people and monitoring of their presence (particularly in crowded situations) in smart indoor environments, specifically concerning buildings that are open to the public (i.e., museums, theatres, libraries, hospitals, public administrative offices, shopping centers and malls, gyms, etc.).

## 6. Conclusions and Future Works

In this paper, an edge-based system for classroom occupancy detection within the context of a university campus was presented. Pictures were taken using Intel RealSense D415 cameras. It was possible to use the simplified version of YOLOv3 by employing transfer learning to fine-tune its weights using images of two types of classrooms (small and large). In this way, it was possible to shift from a classical client–server architecture to a fat client–thin server architecture, in which the prediction was computed directly at the edge. The results showed that the system could be generalized and was able to work correctly for the images of classrooms that were not used for the fine-tuning phase.

As future work, we aim to combine our camera-based approach with other privacy-based sensors, starting with the assumption that an indoor environment is affected by human activities and that the influence can be measured using various sensors. In particular, we are interested in investigating the computation of the density of crowds using carbon dioxide (CO_2_) and particulate matter (PM) sensors, both alone and in combination with our camera-based approach.

## Figures and Tables

**Table 1:** Batch iterations over the course of the training process. For each iteration, the mean average precision (with 50% as the threshold parameter), precision, recall, F1 score and average intersection over union (with a threshold of 0.25) are reported. The best obtained results are reported in bold, corresponding to iteration 65,000.

Batch	mAP@50%	Precision@0.25	Recall@0.25	F1@0.25	AIoU@0.25
1000	17.49	0.61	0.24	0.34	41.27
5000	56.35	0.83	0.57	0.67	64.83
10,000	59.42	0.76	0.63	0.69	59.41
15,000	62.27	0.85	0.6	0.7	67.54
20,000	63.74	0.83	0.63	0.71	64.97
25,000	65.93	0.8	0.6	0.72	63.48
30,000	66.34	0.79	0.67	0.73	63.31
35,000	66.66	0.76	0.69	0.72	60.61
40,000	66.51	0.77	0.69	0.72	60.81
45,000	67.7	0.74	0.71	0.73	59.02
50,000	67.25	0.74	0.7	0.72	59.13
55,000	67.99	0.78	0.69	0.73	62.89
60,000	68.03	0.78	0.69	0.73	62.83
**65,000**	**68.51**	**0.81**	**0.67**	**0.73**	**64.9**
70,000	67.26	0.69	0.73	0.71	54.5
75,000	68.23	0.76	0.71	0.73	60.73
80,000	68	0.75	0.7	0.73	60.51
85,000	67.31	0.72	0.72	0.72	57.54
90,000	68.89	0.74	0.72	0.73	59.11
95,000	68.17	0.74	0.72	0.73	59.45
100,000	67.99	0.74	0.72	0.73	59.36
105,000	67.67	0.74	0.71	0.73	59.6

**Table 2:** Performance of the system using the testing set.

Classroom	Average Accuracy	Standard Deviation	RMSE	MAE
**Small Classrooms**				
1	97.10%	6.78	0.65	0.33
2	96.76%	9.05	0.55	0.21
3	94.07%	13.83	0.62	0.25
4	95.10%	8.58	0.79	0.47
5	95.80%	8.09	2.89	1.22
**Large Classrooms**				
1	95.58%	6.70	2.23	1.14
2	93.44%	15.17	1.93	1.23
3	91.00%	14.76	1.78	1.12

**Table 3:** Performance of the system in large classrooms when evaluating the images that were captured by the two cameras separately.

Large Classrooms				
	**Left Camera**		**Right Camera**	
**Classroom**	**Accuracy**	**Standard Deviation**	**Accuracy**	**Standard Deviation**
1	94.73%	7.43	96.42%	5.80
2	93.91%	17.51	92.97%	12.47
3	90.42%	15.03	91.57%	14.53

## Data Availability

The data presented in this study are available upon request from the corresponding author. The data are not publicly available due to privacy restrictions.

## Abbreviations

The following abbreviations are used in this manuscript:

CSC	Classroom Student Counting
IOU	Intersection over union
FCN	False counting number
LOI	Line of interest
MAE	Mean absolute error
MAP	Mean average precision
PN	Predicted number
R-CNN	Region-based convolutional neural network
RMSE	Root mean square error
RN	Real number
ROI	Region of interest
